# Rehabilitation after surgery for hip fracture – the impact of prompt, frequent and mobilisation-focused physiotherapy on discharge outcomes: an observational cohort study

**DOI:** 10.1186/s12877-024-05206-8

**Published:** 2024-07-23

**Authors:** Daniel Siminiuc, Oya Gumuskaya, Rebecca Mitchell, Jack Bell, Ian D. Cameron, Jamie Hallen, Karen Birkenhead, Sarah Hurring, Brett Baxter, Jacqueline Close, Katie J. Sheehan, Antony Johansen, Mellick J. Chehade, Catherine Sherrington, Zsolt J. Balogh, Morag E. Taylor, Mitchell Sarkies

**Affiliations:** 1 School of Health Sciences, Faculty of Medicine and Health, The University of Sydney, Susan Wakil Health Building, Level 7 D18Western Avenue NSW 2006, Camperdown, Australia; 2 Australian Institute of Health Innovation, Macquarie University, Macquarie Park, NSW 2109 Sydney, Australia; 3 Allied Health Research Collaborative, The Prince Charles Hospital, QLD 4032 Chermside, Australia; 4 Faculty of Medicine and Health, John Walsh Centre for Rehabilitation Research, Kolling Institute, Northern Sydney Local Health Districtand, University of Sydney, NSW 2064 St Leonards, Australia; 5 Neuroscience Research Australia, University of New South Wales, NSW 2031 Randwick, Australia; 6 Implementation Science Academy, Sydney Health Partners, NSW Camperdown, Australia; 7 Te Whatu Ora Waitaha Canterbury, Christchurch, New Zealand; 8 Physiotherapy Department, Princess Alexandra Hospital, QLD 4102 Woolloongabba, Australia; 9 School of Clinical Medicine, University of New South Wales, NSW 2052 Sydney, Australia; 10 Bone & Joint Health, Blizard Institute, Queen Mary University of London, London, UK; 11 School of Medicine, University Hospital of Walesand, Cardiff University, Cardiff, UK; 12 Discipline of Orthopaedics and Trauma, Royal Adelaide Hospital, The University of Adelaide, Adelaide, SA 5005 Australia; 13 Institute for Musculoskeletal Health, The University of Sydneyand, Sydney Local Health District, Gadigal Land, NSW 2006 Sydney, Australia; 14 Sydney School of Public Health and, Faculty of Medicine and Health, Sydney Musculoskeletal Health, The University of Sydney, Gadigal Land, NSW 2006 Sydney, Australia; 15 Department of Traumatology, Trauma and Injury Research Program, John Hunter Hospital and University of Newcastle, Hunter Medical Research Institute, NSW Newcastle, Australia

**Keywords:** Key performance indicator, Walking, Ambulation, Perioperative care, Recovery, Audit, Fracture neck of femur, Clinical quality registry, Orthogeriatric, Physiotherapy

## Abstract

**Purpose:**

To determine the relationship between three postoperative physiotherapy activities (time to first postoperative walk, activity on the day after surgery, and physiotherapy frequency), and the outcomes of hospital length of stay (LOS) and discharge destination after hip fracture.

**Methods:**

A cohort study was conducted on 437 hip fracture surgery patients aged ≥ 50 years across 36 participating hospitals from the Australian and New Zealand Hip Fracture Registry Acute Rehabilitation Sprint Audit during June 2022. Study outcomes included hospital LOS and discharge destination. Generalised linear and logistic regressions were used respectively, adjusted for potential confounders.

**Results:**

Of 437 patients, 62% were female, 56% were aged ≥ 85 years, 23% were previously living in a residential aged care facility, 48% usually walked with a gait aid, and 38% were cognitively impaired prior to their injury. The median acute and total LOS were 8 (IQR 5–13) and 20 (IQR 8–38) days. Approximately 71% (*n* = 179/251) of patients originally living in private residence returned home and 29% (*n* = 72/251) were discharged to a residential aged care facility. Previously mobile patients had a higher total LOS if they walked day 2–3 (10.3 days; 95% CI 3.2, 17.4) or transferred with a mechanical lifter or did not get out of bed day 1 (7.6 days; 95% CI 0.6, 14.6) compared to those who walked day 1 postoperatively. Previously mobile patients from private residence had a reduced odds of return to private residence if they walked day 2–3 (OR 0.38; 95% CI 0.17, 0.87), day 4 + (OR 0.38; 95% CI 0.15, 0.96), or if they only sat, stood or stepped on the spot day 1 (OR 0.29; 95% CI 0.13, 0.62) when compared to those who walked day 1 postoperatively. Among patients from private residence, each additional physiotherapy session per day was associated with a -2.2 (95% CI -3.3, -1.0) day shorter acute LOS, and an increased log odds of return to private residence (OR 1.76; 95% CI 1.02, 3.02).

**Conclusion:**

Hip fracture patients who walked earlier, were more active day 1 postoperatively, and/or received a higher number of physiotherapy sessions were more likely to return home after a shorter LOS.

## Introduction

Hip fractures are a catastrophic injury for older people, responsible for substantial reductions in physical function, and high levels of morbidity and mortality [1]. Even in advanced health systems, approximately half of those with hip fractures do not regain their previous level of function and more than 10% require a change in residence to a residential aged care facility after hip fracture [2–5]. More than 1.66 million hip fractures are estimated to occur annually worldwide with projections that this number will rise to approximately 6 million fractures each year by 2050 [6]. Hip fractures also represent a considerable cost, estimated at over USD $43,669 of health and social care costs per person in the 12 months following injury [7].

One of the key goals of care after hip fracture surgery is to return to walking and the highest possible level of function. The first postoperative days are crucial for recovery, for example, early mobilisation within 1–2 days is recommended after surgery [8, 9] as is thought to accelerate functional recovery [10], is associated with a reduction in the likelihood of death while receiving in-patient hospital care [11, 12], and increases the likelihood of hospital discharge within 30-days postoperatively [13]. Higher frequencies of physiotherapy sessions (three times daily) in acute care have been shown to expedite functional recovery and reduce total hospital length of stay (LOS) by up to 10 days [14]. Furthermore, longer duration (greater than 2 h) of physiotherapy in the first postoperative week is associated with discharge within 30-days [15], discharge home, survival, outdoor mobility recovery, and lower readmission rates [16].

In Australia and the United Kingdom (UK), over 90% of hip fracture patients were offered the opportunity to mobilise by the day after surgery [17, 18], but less than 50% actually walked the day after surgery in Australia [17]. There is limited understanding of the barriers that may hinder the ability to improve rates of day 1 walking postoperatively, such as delirium and dementia, postural hypotension, postoperative anaemia, uncontrolled pain, drowsiness, and process and systems of care [19]. Furthermore, while some patients might not be able to mobilise the first day after surgery, they may succeed another day or achieve other types of activity apart from walking on day 1 (e.g. sitting or standing). It is not clear whether the benefits in hospital discharge outcomes from day 1 walking can be achieved via other types of activity, apart from walking. We sought to address these gaps in the literature by examining the impact of time to first postoperative walk, different levels of day 1 activity, and the frequency of physiotherapy on hospital discharge outcomes.

### Aim

To determine the relationship between three activities: 1) time to first postoperative walk, 2) activity on the day after surgery, and 3) physiotherapy frequency, and hospital LOS and discharge destination after hip fracture.

## Methods

### Study design

A cohort study was conducted from the ANZHFR Acute Rehabilitation Sprint Audit and is reported according to the Strengthening the Reporting of Observational Studies in Epidemiology (STROBE) guidelines. The ANZHFR is a clinical quality registry that collects information on patient demographics, care at presentation, and pre-, peri- and post-operative care, and mortality for 104 public and private hospitals across Australia and New Zealand that provide surgical management for people with a hip fracture [17]. In 2022, the ANZHFR conducted an acute rehabilitation sprint audit to collect additional variables over a defined period on acute rehabilitation practices for patients with hip fracture, with a specific focus on early mobilisation [20]. De-identified data from the audit were linked to routinely collected data in the ANZHFR for 437 surgically managed hip fracture patients across 36 hospitals that opted-in to the audit. In 2022, the ANZHFR included data from 16,395 individual patient records across 97 hospitals. Ethical approval was granted for the ANZHFR in each Australian state and in New Zealand for the ANZHFR and for the sprint audit (except Queensland due to state Public Health Act legislation requirements).

### Data source and participants

To be included in the ANZHFR, a patient must be aged ≥ 50 years, with a hip fracture following a minimal mechanical trauma less than 14 days prior to presentation (including in-hospital fractures). All ANZHFR hospitals (excluding Queensland) were invited to voluntarily take part in the sprint audit. Recruitment of participating hospitals was supported through ANZHFR newsletters and direct invitations.

The protocol was developed in consultation with the Australian Physiotherapy Association, members of the ANZHFR Steering Group and Research Subcommittee, and external collaborators to ensure the data collection provided the most valuable contributions to understand existing care processes and identify opportunities for improvement. The protocol and dataset definitions were developed from a review of the UK’s 2017 physiotherapy ‘hip sprint’ audit [21], hip fracture guidelines [8], and the Australian Commission on Safety and Quality in Health Care’s National Safety and Quality Health Service Standards [9]. The audit questions were piloted at five hospitals before being added to the ANZHFR minimum data set for consecutive, eligible patients admitted from 1st to 30th of June 2022. Data were collected by healthcare providers who routinely collect ANZHFR data from hospital medical records at the participating hospital [22].

### Study exposures

The study exposure variables were: 1) first postoperative walk; 2) type of first day activity; and 3) number of physiotherapy sessions per day. First walk was defined as the first postoperative day the patient was able to walk or step transfer categorised as day 1, day 2 or 3, or day 4 + . First day activity was defined as the type of activity achieved day 1 postoperatively, categorised as walk or step transfer, stood next to the bed or stepped/marched on the spot or sat on the edge of the bed, or transferred with mechanical lifter or no activity achieved. Physiotherapy sessions were defined as the total number of physiotherapist or allied health assistant sessions provided per day for up to seven days during the acute ward admission period. Walking was defined according to the ANZHFR mobilisation definition, as “the patient managed to stand and step transfer out of bed onto a chair/commode or walk. This does not include only sitting over the edge of the bed or standing up from the bed without stepping/walking” [23].

### Study outcomes

The study outcomes included LOS and hospital discharge destination. Reasons for being unable to mobilise day 1 postoperatively were also explored. Hospital LOS was defined as acute (number of inpatient bed days on the acute ward) or total (entire inpatient hospital stay, including acute and subacute care). Discharge destination was defined as discharge to private residence or residential aged care facility/other discharge destination for people who were previously from a private residence. The most common reasons for being unable to mobilise were reported from the patient medical record. Patients whose usual place of residence was “residential aged care” or “other”, those previously non-ambulant prior to their hospital admission and those experiencing in-hospital death were excluded from the discharge destination analysis. Patients who were previously non-ambulant prior to their hospital admission and those experiencing in-hospital death were excluded from the LOS analyses.

### Potential confounders

Potential confounders were entered into the analysis as covariates measured according to the ANZHFR [23], identified as those considered clinically relevant and where previous research has indicated an association with the study exposures or study outcomes. These included age (50-84y vs 85y +), usual place of residence (private residence vs residential aged care facility/other), pre-admission walking ability (with or without an aid), pre-admission cognitive state (impaired or not impaired), ASA grade (1/2, 3, or 4), time to surgery (≤ 48 h vs > 48 h), type of fracture (intra vs extracapsular), and type of anaesthesia (general, spinal/ regional, or general and spinal/regional) [19, 24]. Some variable categories were collapsed due to low patient numbers (e.g. ASA grade 1/2) and the age cutoff was used in recognition of potentially poorer outcomes for older adults aged 85 years and older [25].

### Data analysis

Patients with complete data for exposures, outcomes, and potential confounders were included in the analyses. Patient demographics were described using frequencies and percentages. Outcomes were summarised descriptively using frequencies and percentages for discharge destination and median and interquartile range (IQR) for LOS. Chi-square tests of independence were used to compare patient demographics for older adults with a hip fracture by day 1 mobilisation rates postoperatively. Generalised linear regression was used to calculate coefficients and 95% confidence intervals (CIs) for the association between each exposure and acute ward and total hospital LOS. Logistic regression was used to calculate odds ratios (OR) and 95% CIs for the association between each exposure and discharge destination. Both crude models (including exposure and outcome only) and adjusted models (include exposure, outcome, and all covariates) were applied to each exposure and analysed outcome. Models were checked for multicollinearity and specification and statistical significance was set at p ≤ 0.05. All statistical analyses were undertaken using STATA (StataCorp. (2023). Stata Statistical Software: Release 18. College Station, TX: StataCorp LLC).

The number of physiotherapy sessions and discharge destination analysis model was not linear, so a logarithmic transformation was applied to the physiotherapy sessions exposure variable as a sensitivity analysis. The transformed model exhibited linearity providing a more satisfactory model fit and was reported along with the non-logarithmic model.

## Results

### Patient characteristics

**Fig. 1:**
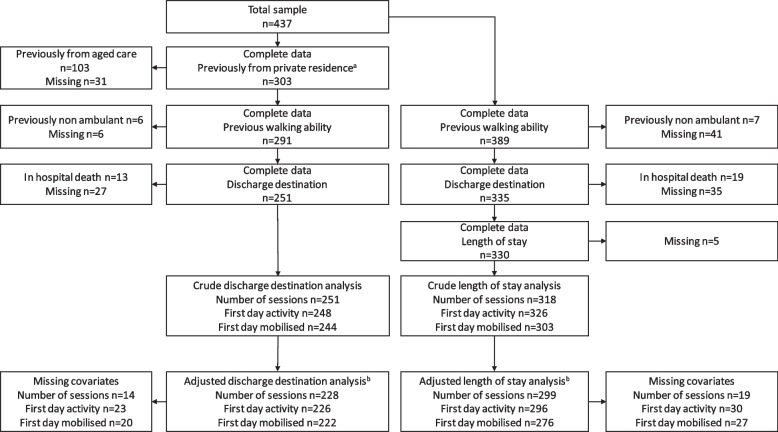
Study flowchart count of exposure and outcome. ^a^Includes unit in a retirement village. ^b^ Excludes missing observations from confounders

Initial exposure and outcome data were available for a total of 437 patients. Of these, 62% (*n* = 270/437) were female, 46% (*n* = 201/437) were aged ≥ 85 years, 23% (*n* = 102/437) were previously living in a residential aged care facility, 48% (*n* = 211/437) usually walked with a gait aid, and 38% (*n* = 166/437) were cognitively impaired prior to their hip fracture (Table 1). A total of 35% (*n* = 151/437) patients mobilised day 1 postoperatively and the most common reasons for being unable to mobilise were: 29% (*n* = 64/151) delirious, agitated, confused or drowsy, 22% (*n* = 48/151) haemodynamic instability, 14% (*n* = 30/151) inadequate pain control, 7% (*n* = 15/151) anaemia, and 6% (*n* = 14/151) refused (Table 2). Following exclusions, 80% (*n* = 251/315) of cases had complete data for discharge destination and 80% (*n* = 330/411) for hospital LOS (Fig. 1).

**Table 1:** Patient demographics

	**Total** *n* = 437^†^	**Mobilised day 1** *n* = 184	**Not mobilised day 1** *n* = 253	**χ**^**2**^** (df)** *p* value^§^
	*n* (%)	*n* (%)	*n* (%)	
Sex				
Male	135 (30.9)	62 (33.7)	73 (28.9)	7.30 (2) *p* = 0.026
Female	270 (61.8)	114 (62.0)	156 (61.7)	
Not known	32 (7.3)	8 (4.3)	20 (7.9)	
Age				
50–84	202 (46.2)	106 (57.6)	96 (37.9)	14.5 (2) *p* < 0.001
85 +	201 (46.0)	69 (37.5)	132 (52.2)	
Not known	34 (7.8)	9 (4.9)	21 (8.3)	
Usual Place of Residence				
Private Residence^a^	300 (68.6)	144 (78.3)	156 (61.7)	11.94 (2) *p *= 0.003
Residential Aged Care Facility^b^	102 (23.3)	30 (16.3)	72 (28.5)	
Not known	35 (8.0)	10 (5.4)	21 (8.3)	
ASA Grade^c^				
1–2	53 (12.1)	29 (15.8)	24 (9.5)	14.34 (4) *p* = 0.006
3	219 (50.1)	99 (53.8)	120 (47.4)	
4	86 (19.7)	29 (15.8)	57 (22.5)	
Not known	79 (18.1)	27 (14.7)	48 (19.0)	
Pre-Fracture Walking Ability				
Without aid	174 (39.8)	89 (52.4)	85 (33.6)	6.82 (2) *p* = 0.033^‡‡^
With aid^d^	211 (48.3)	80 (47.1)	131 (51.8)	
Non ambulant	7 (1.6)	1 (0.6)	6 (2.4)	
Not known	45 (10.3)	14 (7.6)	27 (10.7)	
Cognitive status prior to admission				
Normal cognition	224 (51.3)	113 (61.4)	111 (43.9)	12.51 (2) *p* = 0.002
Impaired cognition	166 (38.0)	56 (30.4)	110 (43.5)	
Not known	47 (10.8)	15 (8.2)	28 (11.1)	
Time to surgery^e^				
Surgery completed within 48 h	292 (66.8)	130 (70.7)	162 (64.0)	4.37 (2) *p* = 0.113
Surgery completed after 48 h	105 (24.0)	41 (22.3)	64 (25.3)	
Not known	40 (9.2)	13 (7.1)	23 (9.1)	
Type of fracture				
Intracapsular^f^	199 (45.5)	96 (52.2)	103 (40.7)	4.52 (2) *p* = 0.105
Intertrochanteric/ subtrochanteric^g^	196 (44.9)	74 (40.2)	122 (48.2)	
Not known	42 (9.6)	14 (7.6)	24 (9.5)	
Type of anaesthesia				
General	221 (50.6)	92 (50.0)	129 (51.0)	1.44 (4) *p* = 0.837
Spinal/ regional	63 (14.4)	31 (16.9)	32 (12.6)	
General and spinal/ regional	104 (23.8)	44 (23.9)	60 (23.7)	
Not known	49 (11)	17 (9.2)	28 (11.1)	

^a^Includes unit in a retirement village

^b^Includes other previous residence not considered private residence

^c^ASA grades patients into the following five categories: 1, healthy patient; 2, patient with mild systemic disease; 3, patient with severe systemic disease; 4, patient with incapacitating systemic disease; 5, moribund patient who is not expected to survive beyond 24 h without the relevant operation (no ASA grade 5 in sample)

^d^Usually walks with either a stick, crutch or two aids or frame (with or without assistance of a person)

^e^From the time of arrival in the emergency department of the first hospital, or diagnosis of a fracture if the fracture occurred as an inpatient

^f^Includes intracapsular undisplaced/impacted displaced and intracapsular displaced

^g^Includes per/intertrochanteric and subtrochanteric

^†^Does not include patients with missing data for day 1 mobilisation

^§^Excludes missing categories

^‡‡^Excludes non-ambulant category

**Table 2:** Barriers to first day mobilisation postoperatively

	**Total** *n* = 269
	*n* (%)
Delirium, agitated, confused or drowsy	64 (23.8)
Low or high blood pressure, tachycardia, or bradycardia	48 (17.8)
Pain inadequately controlled	30 (11.2)
Anaemia (low haemoglobin)	15 (5.6)
Patient refused	14 (5.2)
Bedbound pre-admission	7 (2.6)
Awaiting imaging or weight bearing status	5 (1.9)
Inadequate physiotherapy staffing	4 (1.5)
Patient not available	2 (0.7)
Non weight bearing	1 (0.4)
Other	30 (11.2)
Not known	49 (18.2)

### Length of stay

Hip fracture patients had a median acute ward LOS of 8 days (5–13 IQR) and a median total hospital LOS of 20 days (8–38 IQR). Crude and adjusted odds ratios for the association between 1) first walk, 2) first day activity and 3) physiotherapy sessions; and length of stay are presented in Table 3. Following adjustment for covariates, day of first walk and day 1 activity were not associated with acute ward LOS; however, each additional physiotherapy session per day was associated with a -2.2 (95% CI -3.3, -1.0) day shorter acute ward LOS. Patients who walked day 2–3 had a 10.3 (95% CI 3.2, 17.4) day longer total hospital LOS compared to those who walked day 1 after surgery. The patients who only managed to transfer with a mechanical lifter or did not get out of bed had a 7.6 (95% CI 0.6, 14.6) day longer total hospital LOS, compared to those who walked as their first day activity. There was no association between the number of physiotherapy sessions per day and total hospital LOS (*p* = 0.052).

**Table 3:** Associations between acute rehabilitation activities and hospital length of stay

		**Acute length of stay**^**a**^					**Total length of stay**^**b**^				
	**Total patients**	**Acute length of stay**	**Crude**		**Adjusted**		**Total length of stay**	**Crude**		**Adjusted**	
	*n* (%)	**Median (IQR)**	**Coefficient (95% CI)**	*p* value	**Coefficient (95% CI)**	*p* value	**Median (IQR)**	**Coefficient (95% CI)**	*p* value	**Coefficient (95% CI)**	*p* value
First walk^c^ Crude *n* = 303 Adjusted *n* = 276											
Day 1	151 (49.8)	7 (4–12)	0^b^		0^b^		16 (7–34)	0^b^		0^b^	
Day 2–3	60 (19.8)	**9 (6–15)**	**2.37 (-0.29, 5.02)**	**0.080**	**1.91 (-0.88, 4.71)**	**0.178**	**28 (13–49)**	**10.19 (2.79, 17.60)**	**0.007**	**10.29 (3.16, 17.43)**	**0.005**
Day 4 +	40 (13.2)	**11 (7–16)**	**3.69 (0.60, 6.79)**	**0.019**	**1.74 (-1.58, 5.06)**	**0.304**	**31 (18–48)**	**11.02 (2.39, 19.35)**	**0.013**	**7.06 (-1.42, 15.55)**	**0.102**
Did not walk	52 (17.2)	**7 (5–12)**	**-0.21 (-3.01, 2.58)**	**0.881**	**0.39 (-2.93, 3.72)**	**0.816**	**10 (5–26)**	**-4.00 (-11.81, 3.80)**	**0.313**	**0.91 (-7.58, 9.41)**	**0.832**
First day activity^c^ Crude *n* = 326 Adjusted *n* = 296											
Walk or step transfer^d^	151 (46.3)	7 (4–12)	0^b^		0^b^		16 (7–34)	0^b^		0^b^	
Sitting/ Standing/ Stepping^e^	92 (28.2)	**9 (6–14)**	**1.16 (-1.13, 3.45)**	**0.320**	**1.27 (-1.12, 3.67)**	**0.295**	**21 (10–39)**	**4.55 (-2.11, 11.21)**	**0.180**	**4.95 (-1.57, 11.47)**	**0.095**
Mechanical lifter/ None	83 (25.5)	**9 (5–16)**	**2.08 (-0.29, 4.45)**	**0.085**	**1.54 (-1.04, 4.11)**	**0.241**	**21 (7–42)**	**5.45 (-1.43, 12.34)**	**0.120**	**7.59 (0.59, 14.60)**	**0.034**
Physiotherapy sessions^c,f^ Crude *n* = 299 Adjusted *n* = 299											
Daily sessions	**330 (100.0)**	**8 (5–13)**	**-1.79 (-2.91, -0.67)**	**0.002**	**-2.15 (-3.34, -0.96)**	** < 0.001**	**20 (8–38)**	**-0.56 (-3.96, 2.84)**	**0.748**	**-3.36 (-6.75, 0.03)**	**0.052**

^a^Length of stay in the acute ward postoperatively

^b^Total hospital length of stay days (including acute and sub-acute admission)

^c^Postoperatively

^d^Walked means the patient managed to stand and step transfer out of bed onto a chair/commode or walk. This does not include only sitting over the edge of the bed or standing up from the bed without stepping/walking

^e^Includes sitting on edge of bed, sit to stand (standing), and stepping/marching on the spot

^f^Average number of physiotherapy or allied health assistant sessions per day for up to the first seven days

0^b^Reference category used during analysis

The following covariates were adjusted for age (50-84y vs 85y +), usual place of residence (private residence vs residential aged care facility/other), pre-admission walking ability (with or without an aid), pre-admission cognitive state (impaired or not impaired), ASA grade (1/2, 3, or 4), time to surgery (≤ 48 h vs > 48 h), type of fracture (intra vs extracapsular), and type of anaesthesia (general, spinal/ regional, or general and spinal/regional)

*CI* = confidence interval, *IQR* = interquartile range

### Discharge destination for previously ambulant people from private residences

Seventy-one percent (*n* = 179/251) of patients were discharged from hospital to private residences and 29% (*n* = 72/251) were discharged to a residential aged care facility. Crude and adjusted odds ratios for the association between 1) first walk, 2) first day activity and 3) physiotherapy sessions; and discharge destination are presented in Table 4. Following adjustment for covariates, patients who walked day 2–3 (OR 0.38; 95% CI 0.17, 0.87) or day 4 + (OR 0.38; 95% CI 0.15, 0.96) had a reduced odds of being discharged to private residence compared to those who mobilised day 1 after surgery. Patients who only managed sitting/standing/stepping (OR 0.29; 95% CI 0.13, 0.62) were less likely to be discharge to private residence compared to those who walked as their first day activity; and each additional physiotherapy session per day was associated with a 76% increased odds of being discharged to private residence.

**Table 4:** Associations between acute rehabilitation processes and discharge destination for patients previously living in private residence

		**Discharge destination**		**Discharge to private residence**^**a**^			
	**Total patients**	**Private residence**^**b**^	**Residential aged care facility**	**Crude**		**Adjusted**	
	*n* (%)	*n* (%)	*n* (%)	**OR (95% CI)**	*p* value	**OR (95% CI)**	*p* value
First walk^c^ Crude *n* = 244 Adjusted *n* = 222							
Day 1	128 (52.5)	106 (82.8)	22 (17.2)	0^b^			0^b^
Day 2–3	58 (23.7)	38 (65.5)	20 (34.5)	0.39 (0.19, 0.80)	0.010	0.38 (0.17, 0.87)	0.022
Day 4 +	40 (16.4)	22 (55.0)	18 (45.0)	0.25 (0.12, 0.55)	0.001	0.38 (0.15, 0.96)	0.041
Did not walk	18 (7.4)	9 (50.0)	9 (50.0)	0.21 (0.07, 0.58)	0.003	0.32 (0.09, 1.10)	0.070
First day activity^c^ Crude *n* = 248 Adjusted *n* = 226							
Walk or step transfer^d^	128 (51.6)	106 (82.8)	22 (17.19)	0^b^			0^b^
Sitting/ Standing/ Stepping^e^	68 (27.4)	40 (58.8)	28 (41.2)	0.30 (0.15, 0.58)	< 0.001	0.29 (0.13, 0.62)	0.001
Mechanical lifter/ None	52 (21.0)	30 (57.7)	22 (42.3)	0.28 (0.14, 0.58)	0.001	0.46 (0.19, 1.10)	0.083
Physiotherapy sessions^c,f^ Crude *n* = 251 Adjusted *n* = 228	**Median (IQR)**	**Median (IQR)**	**Median (IQR)**	**OR (95% CI)**	*p* value	**OR (95% CI)**	*p* value
Daily sessions	0.85 (0.57, 1.14)	0.86 (0.57, 1.14)	0.71 (0.43, 1.14)	1.52 (0.96, 2.40)	0.075	1.57 (0.96, 2.58)	0.074
Daily sessions log^g^	-0.16 (-0.56, 0.13)	-0.15 (-0.56, 0.13)	-0.34 (-0.85, 0.13)	1.67 (1.05, 2.65)	0.031	1.76 (1.02, 3.02)	0.042

^a^Reference category is residential aged care facility/other

^b^Includes unit in a retirement village

^c^Postoperatively

^d^Walked means the patient managed to stand and step transfer out of bed onto a chair/commode or walk. This does not include only sitting over the edge of the bed or standing up from the bed without stepping/walking

^e^Includes sitting on edge of bed, sit to stand (standing), and stepping/marching on the spot

^f^Average number of physiotherapy or allied health assistant sessions per day for up to the first seven days

^g^logarithmic model

0^b^Reference category used during analysis

The following covariates were adjusted for: age (50-84y vs 85y +), usual place of residence (private residence vs residential aged care facility/other), pre-admission walking ability (with or without an aid), pre-admission cognitive state (impaired or not impaired), ASA grade (1/2, 3, or 4), time to surgery (≤ 48 h vs > 48 h), type of fracture (intra vs extracapsular), and type of anaesthesia (general, spinal/ regional, or general and spinal/regional)

*CI = confidence interval, IQR = interquartile range, OR = odds ratio*

## Discussion

This study demonstrates that previously ambulant hip fracture patients had a shorter acute ward LOS if they received more physiotherapy sessions and had a shorter total hospital LOS if they walked earlier and were more active on their first day postoperatively. Older adults previously living in private residence were less likely to be discharged to a residential aged care facility if they walked earlier, were more active on their first day postoperatively, and received more physiotherapy sessions.

A common goal for patients previously living in a private residence is to return home, making this a valuable outcome measure that is important for patients and healthcare professionals. In Ireland, patients who mobilised the first day after surgery were 24% more likely to be discharged to private residence [26]. However, the Irish study did not provide an indication of whether older adults previously living in private residences returned to their home. In contrast, our study excluded people living in a residential aged care facility prior to their hip fracture thus, enabling us to demonstrate that people who mobilised earlier and more frequently postoperatively were more likely to return home. Previous qualitative research has highlighted that returning home and returning to previous activities is one of the most frequently reported short-term rehabilitation goals after hip fracture [27]. Therefore, efforts should be directed to not only offering hip fracture patients the opportunity to walk the day of, or day after, surgery but also facilitating higher rates of actual walking day 1.

Despite clinical practice guideline recommendations indicating the clear benefits of walking day 1 postoperatively [8, 9], there are many people who are unable to achieve this due to barriers reported in our study and other published literature (e.g. confusion, low haemoglobin, or nausea and vomiting) [19]. These barriers to day 1 walking are potentially preventable or amenable through perioperative interventions that optimise patients medically (e.g. orthogeriatric review) and processes and systems of care (e.g. timely surgery). Previous research has shown that providing exercises in addition to walking does not improve the probability of hospital discharge within 30 days [15]; however, recipients of exercise have been shown to have higher probabilities of being discharged home, and of survival, recovery of outdoor mobility, and lower readmission rates [16]. Our study sought to explore the impact of other types of day 1 activity for older adults unable to walk. We found adults only achieving sitting, standing or stepping on day 1 were less likely to return home compared to adults who walked, further emphasising the importance of implementing perioperative interventions that can overcome the barriers to day 1 walking. A recent systematic review identified several perioperative interventions that could improve rates of day 1 walking by preventing or addressing barriers, including pathways and models of care, early surgery within six hours of diagnosis, direct clinical supervision of physiotherapists, and multidisciplinary rehabilitation approaches [28].

Our study identified that walking day 2–3 instead of day 1 postoperatively and only transferring with a mechanical lifter or not getting out day 1 led to a 7–11 day increase in total hospital LOS. This study also identified lower acute ward LOS from higher frequencies of physiotherapy sessions. A non-significant association was observed for the frequency of physiotherapy sessions and total hospital length of stay These findings are somewhat concordant with a previous trial by Kimmel et al., who demonstrated up to 10 day reductions in total hospital LOS for patients receiving high intensity physiotherapy after hip fracture [14]. A patient’s LOS potentially provides an indication of whether recovery is accelerated or delayed after surgery and longer hospital stays increase risk of hospital acquired complications [22]. However, the use of LOS as an outcome is challenging, as LOS can be influenced by factors other than a person’s functional status [29].

### Limitations

This study had some limitations: 1) the coverage of ANZHFR hospitals in Australia was not 100% at the time of this study and it is not known whether there are systematic differences in the hospital volunteering to participate in the Audit compared to those that did not participate; 2) approximately 20% of patients were missing from the discharge destination and length of stay analysis; 3) it is possible that there was incomplete collection of potential confounders (e.g. concurrent associated injuries); and considering the relatively small sample size the results may need to be considered as hypothesis generating; and 4) data from Australian and New Zealand hospitals may have limited generalisability to other settings. As with any observational study, it is difficult to draw causal inferences regarding the direction of the relationships observed as healthier patients may be more likely to mobilise and participate in more physiotherapy sessions.

### Future directions

Future research could consider pragmatic strategies to implement hip fracture clinical practice guidelines for acute rehabilitation into routine care. The average number of physiotherapy sessions per day was 0.85, in contrast to clinical practice guidelines which recommend at least once per day [8, 9]. Also, there was substantial variation in day 1 mobilisation rates between participating sites, indicating potential room for improvement. Barriers to day 1 walking were predominantly related to patient characteristics that could be preventable or amenable to perioperative interventions to ensure patient optimisation (e.g. adequate pain relief, timely surgery, and orthogeriatric review). Some of these interventions can be delivered by allied health professionals and others must be coordinated with the multidisciplinary team. Barriers have been previously identified to the implementation of change to allied health clinical practice, such as attitudes towards evidence, skills in critical appraisal, and limited authority to promote change [30]. Knowledge brokering has been explored as a potential approach to overcome these barriers and promote evidence-informed resource allocation decisions by allied health managers [31, 32] and video knowledge translation strategies have been shown to improve allied health understanding of evidence [33]. Our findings are confirmatory of other physiotherapy focussed audits in hip fracture care, providing potential indicators that could be collected as part of routine registry measures. Future research could consider what additional rehabilitation indicators could be measured in future audits as improvements in rehabilitative care for hip fracture patients evolves.

## Conclusion

This study provides novel insights into the relationship between different acute rehabilitation approaches, focussed on walking and physiotherapy, and hospital discharge outcomes for hip fracture patients. Earlier walking, more day 1 activity, and higher frequency of physiotherapy sessions was associated with a greater likelihood of returning to private residence with a shorter LOS. We identified that each day walking is delayed (e.g. day 2–3 and day 4 +) and day 1 activities that don’t include walking (sitting/standing/stepping or mechanical lifter/none) lead to poorer hospital discharge outcomes. These insights highlight the importance of optimising perioperative care processes to ensure more hip fracture patients can walk day 1 postoperatively.

## Data Availability

The datasets generated and/or analysed during the current study are available in the Australian and New Zealand Hip Fracture Registry, https://anzhfr.org/. Ethical approval was granted for the ANZHFR in each Australian state and in New Zealand for the ANZHFR and the sprint audit (except Queensland due to state Public Health Act legislation requirements), including an approved waiver of consent.
